# NLRP3/IL‐1β induced myeloid‐derived suppressor cells recruitment and PD‐L1 upregulation promotes oxaliplatin resistance of hepatocellular carcinoma

**DOI:** 10.1002/mco2.447

**Published:** 2023-12-19

**Authors:** Wenfeng Liu, Feng Zhang, Bing Quan, Fan Yao, Rongxin Chen, Zhenggang Ren, Xin Yin

**Affiliations:** ^1^ Department of Hepatic Oncology, Liver Cancer Institute, Zhongshan Hospital Fudan University Shanghai China; ^2^ Department of National Clinical Research Center for Interventional Medicine Zhongshan hospital, Fudan university Shanghai China

**Keywords:** hepatocellular carcinoma, myeloid‐derived suppressor cells, NLRP3/IL‐1β, oxaliplatin resistance, PD‐L1

## Abstract

Oxaliplatin is commonly used as the first‐line chemotherapeutic agent for advanced hepatocellular carcinoma (HCC). Unfortunately, the acquired resistance, limits the effectiveness of oxaliplatin and the underlying mechanisms remain unknown. Therefore, we explored the role of NOD‐like receptor protein 3 (NLRP3)/IL‐1β in mediating oxaliplatin resistance in HCC. We observed that NLRP3/IL‐1β expression was much higher in oxaliplatin‐resistant HCC cells. To further understand its impact on drug resistance, we knocked down NLRP3 and observed that it sensitized HCC cells to the growth‐inhibitory effects of oxaliplatin and induced cell apoptosis. NLRP3/IL‐1β overexpressing tumor cells also attracted polymorphonuclear myeloid‐derived suppressor cells. Using mouse models, we demonstrated that NLRP3/IL‐1β inhibition by short hairpin RNA or MCC950 effectively overcame oxaliplatin resistance. Furthermore, NLRP3/IL‐1β inhibition resulted in reduced expression of PD‐L1. We also found that PD‐L1 antibody combined with NLRP3/IL‐1β blockade displayed significant antitumor effect in HCC. Overall, our study provides compelling evidence supporting the essential role of NLRP3/IL‐1β in conferring resistance to oxaliplatin and reshaping the immunosuppressive microenvironment in HCC. Targeting NLRP3/IL‐1β presents a potential therapeutic target for overcoming oxaliplatin resistance and reshaping microenvironment of HCC.

## Graphical abstract

The study shows that NLRP3 is upregulated in oxaliplatin‐resistant HCC cells and has a tumor‐intrinsic function in generating an immunosuppressive hepatocellular carcinoma environment by activating IL‐1β and PD‐L1 upregulation. The combination with PD‐L1 and IL‐1β blockades could represent a promising antitumor strategy for oxaliplatin‐resistant HCC

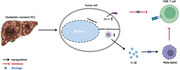

## 1 INTRODUCTION

Based on the Global Cancer Statistics 2022, primary liver cancer (PLC) ranks seventh in incidence among all malignant tumors.^1^ Owing to its high malignancy and rapid progression, it ranks second in terms of mortality rate.^2^ Hepatocellular carcinoma (HCC) is the primary type of PLC, accounting for approximately 75−85%. Surgical resection, transcatheter arterial chemoembolization (TACE), radiofrequency ablation, systemic chemotherapy, targeted drugs, and immunotherapy are available treatment options for HCC.^3^, ^4^ However, there are still challenges such as postoperative recurrence, development of drug resistance, and limited long‐term efficacy.

Oxaliplatin is the most commonly used chemotherapeutic drug for TACE. And oxaliplatin also serves as the foundation of the adjuvant oxaliplatin, fluorouracil, and leucovorin chemotherapy regimen, which is the first‐line chemotherapy for HCC patients. Nevertheless, the development of inherent or acquired chemoresistance in tumors poses a significant obstacle to the effectiveness of platinum‐based treatments. Tumor growth is a dynamic process that involves intricate interactions between tumor cells and their surrounding environment, especially when exposed to the pressures exerted by chemotherapeutic drugs.^5^, ^6^ Recent advancements have revealed that combining chemotherapy with drugs targeting the tumor microenvironment (TME) can help overcome drug resistance.^7^, ^8^ Therefore, therapies focusing on the TME present a promising and innovative approach to address oxaliplatin insensitivity.

Evading immune surveillance is a prominent characteristic of HCC, and it is primarily driven by the recruitment of immunosuppressive cells. Myeloid cells are the predominant cell type in hematopoiesis and play a crucial role in adaptive immunity.^9^, ^10^ Emerging evidence has indicated that myeloid cells can be converted into immunosuppressive cells. Among these cells, myeloid‐derived suppressor cells (MDSCs) have been identified as a population of immature myeloid cells that significantly expand and serve as negative regulators of immune responses.^11^, ^12^, ^13^ MDSCs can be further divided into polymorphonuclear MDSCs (PMN‐MDSCs) and monocyte MDSC (M‐MDSCs).^14^ Previous research has emphasized the significant contribution of MDSCs in the development of HCC.^15^, ^16^ In our previous study, we observed that PMN‐MDSCs were highly enriched in oxaliplatin resistant HCC.^17^ However, there is little research to date that explores the mechanisms through which MDSCs regulate the chemoresistance of HCC.

NOD‐like receptor protein 3 (NLRP3), a member of the canonical inflammasome family of leucine‐rich repeat proteins, plays a crucial role in cancer pathogenesis by regulating apoptotic proteins and immune responses.^18^, ^19^ Activation of NLRP3 has been observed in various human tumors. It has been reported that NLRP3 activation can lead to increased IL‐1β levels and promote metastasis in HCC.^20^ Furthermore, mice that received intravenous implantation of NLRP3‐deficient HCC cells exhibited significantly reduced tumor growth and lower rates of metastasis compared with mice implanted with normal HCC cells. The loss of NLRP3 could inhibit MHC class I polypeptide‐related sequence A (MICA) exfoliation of HCC cells and increase the probability of natural killer group 2, member D–MICA interaction, thereby increasing the sensitivity of HCC to natural killer cell killing.^21^ In previous study, expression and activation of NLRP3 inflammasome was significantly increased in oral squamous cell carcinoma tissues of patients who received 5‐fluorouracil‐based chemotherapy.^22^ Moreover, inhibition of NLRP3 inflammasome activation by andrographolide sulfonate also contributes to 5‐fluorouracil sensitization.^23^ Additionally, it has been found that another chemotherapy drug, cisplatin, can activate NLRP3 in tumor cells, and targeting NLRP3 may overcome chemoresistance.^24^ However, limited research has investigated the relationship between NLRP3 and chemotherapy resistance in HCC. So we want to explore the relationship between oxaliplatin (the most commonly used chemotherapeutic drug) and NLRP3 level and the role of NLRP3 in of oxaliplatin sensitivity in HCC.

## 2 RESULTS

### 2.1 Oxaliplatin induced NLRP3 and IL‐1β expression in vitro and in vivo

**FIGURE 1:**
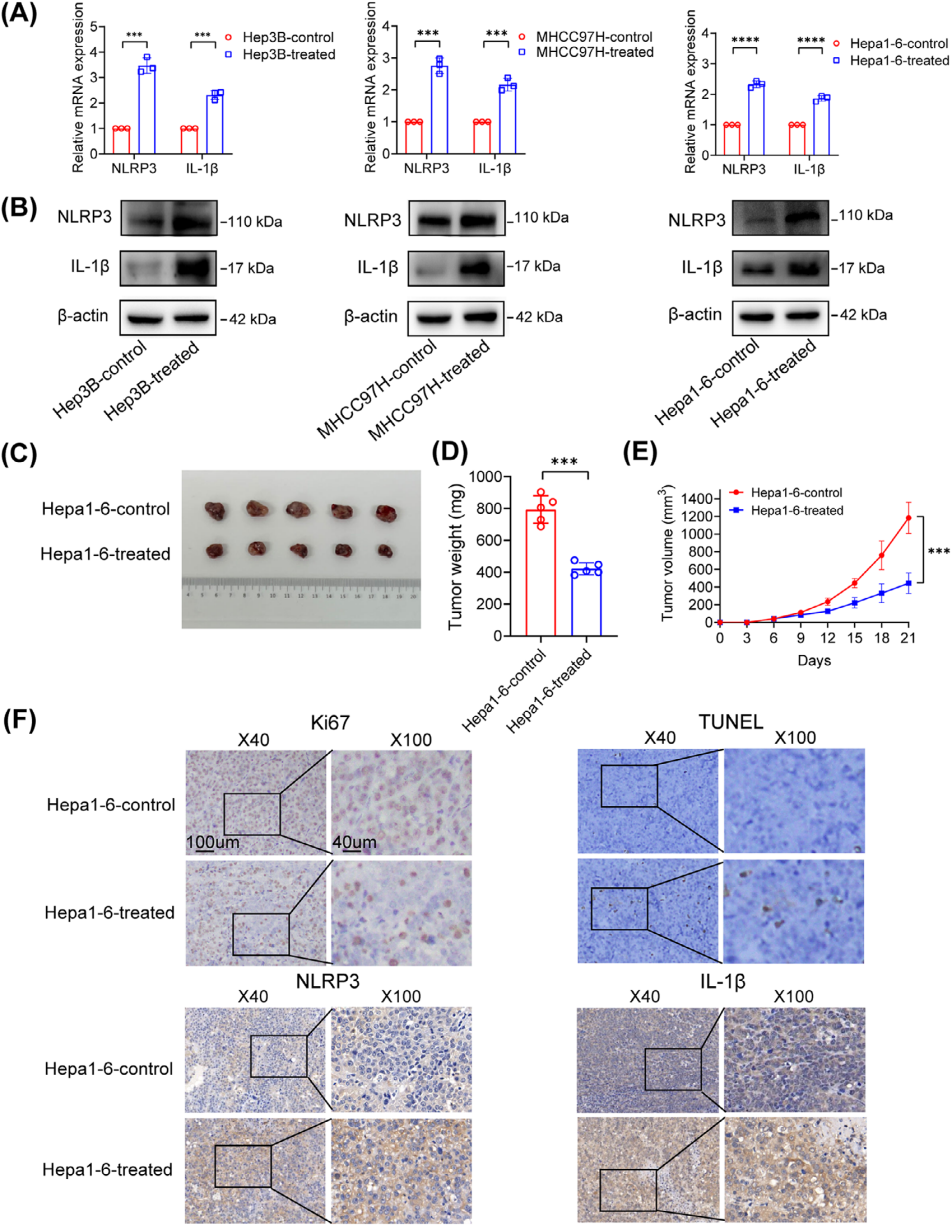
Oxaliplatin induced NLRP3 and IL‐1β expression in vitro and in vivo. (A and B) The mRNA and proteins levels of NLRP3 and IL‐1β in HCC cells treated with or without oxaliplatin. (C and D) The appearance and growth curves of the subcutaneous tumors. (E) Tumor weight was analyzed between different groups. (F) Representative pictures of IHC staining using Ki67 and TUNEL as markers of proliferation and apoptosis in the tumor tissues. *p < 0.05, **p < 0.01, ***p < 0.001.

Multiple signaling pathways were activated in oxaliplatin‐resistant HCC cells. To study the relationship between NLRP3 and chemotherapy response, Hep3B, MHCC97H, and Hepa1‐6 were treated with 50 μM oxaliplatin for 48 h. Quantitative real‐time polymerase chain reaction (qPCR) and western blot analyses showed that the NLRP3 and IL‐1β expression increased significantly after oxaliplatin treatment (Figures 1A and B). This phenomenon was also observed in a mouse model of HCC. We established a subcutaneous tumor mouse model using Hepa1‐6 cells and observed that administration of oxaliplatin had a pronounced effect on inhibiting tumor growth and led to notable upregulation of NLRP3 and IL‐1β. Additionally, oxaliplatin treatment decreased Ki67 expression and increased cell apoptosis (Figures 1C and F).

### 2.2 NLRP3 was highly expressed in oxaliplatin‐resistant HCC cells and involved in oxaliplatin resistance

**FIGURE 2:**
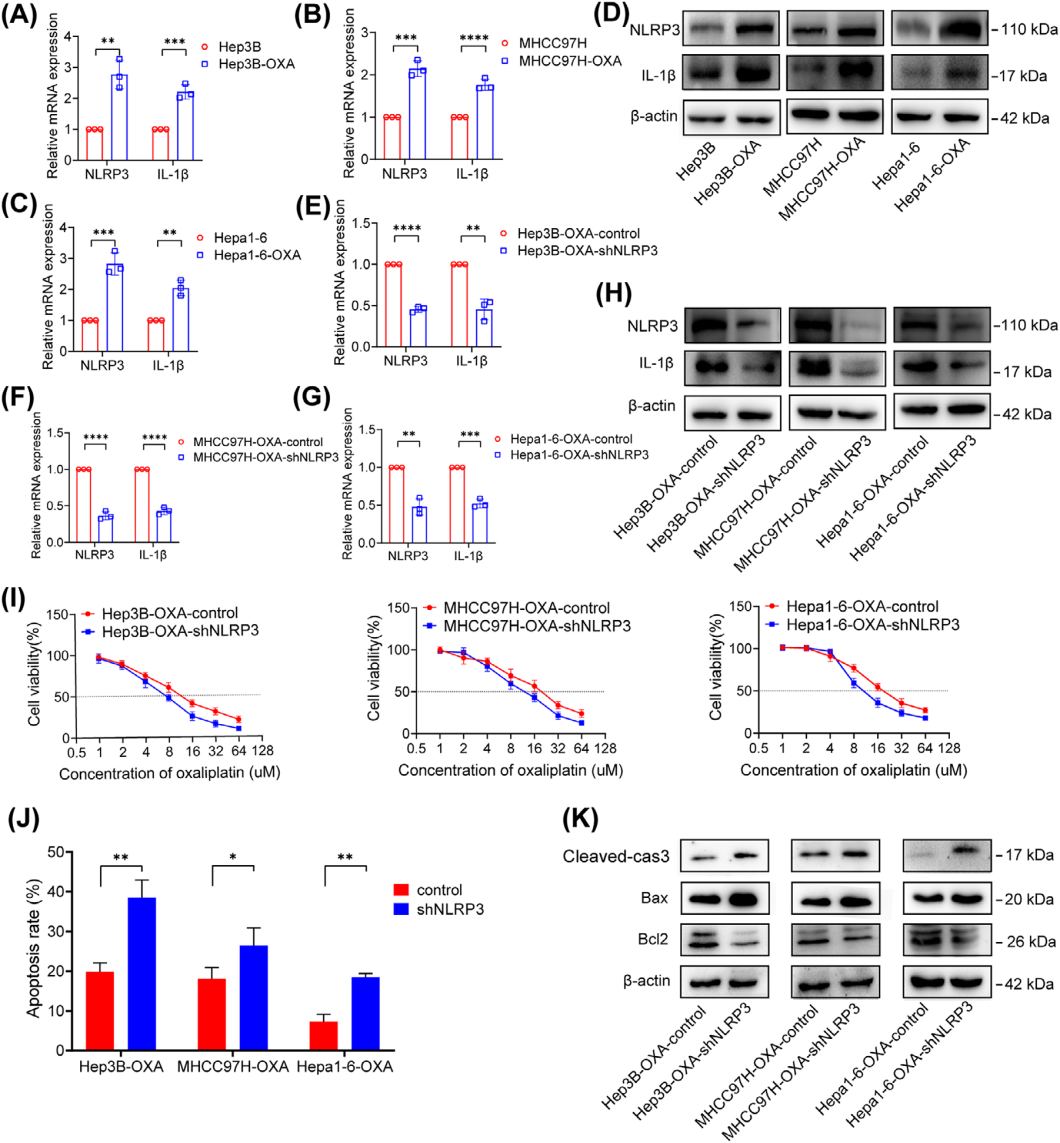
NLRP3 was highly expressed in oxaliplatin‐resistant HCC cells and involved in oxaliplatin resistance. (A–C) The mRNA expression of NLRP3 and IL‐1β in HCC cells and oxaliplatin‐resistant HCC (Hep3B‐OXA, MHCC97H, and Hepa1‐6‐OXA) cells. (D) NLRP3 and IL‐1β proteins in HCC and oxaliplatin‐resistant HCC cells were detected. (E–G) Hep3B‐OXA, MHCC97H, and Hepa1‐6‐OXA cells were transfected with scramble shRNA and lentiviral shRNA targeting NLRP3. RT‐PCR confirmed the downregulation of NLRP3 and L‐1β. (H) NLRP3 and IL‐1β proteins in oxaliplatin‐resistant HCC cells after NLRP3 silencing. (I) The IC50 values of oxaliplatin‐resistant HCC cells transfected with NLRP3 shRNA were significantly lower than those transfected with control lentiviral vectors. (J) The proapoptotic effect of NLRP3 silencing on oxaliplatin‐induced apoptosis was confirmed by flow cytometry in oxaliplatin‐resistant HCC cells. (K) Cleaved‐cas3, bcl2, and bax proteins were examined in different group. *p < 0.05, **p < 0.01, ***p < 0.001.

We established Hep3B‐OXA, MHCC97H‐OXA, and Hepa1‐6‐OXA cell lines using previously described methods. Compared with Hep3B, MHCC97H, and Hepa1‐6 cells, we observed an upregulation of NLRP3 and IL‐1β in the oxaliplatin‐resistant strains (Figures 2A–D). The knockdown effect of NLRP3 using short hairpin RNA (shRNA) was validated at the mRNA and protein levels, respectively (Figures 2E–H). The half‐maximal inhibitory concentration (IC50) of oxaliplatin was significantly reduced in the cells after NLRP3 knockdown (Figure 2I). We also found that the knockdown of the NLRP3 by shRNA promoted the proapoptotic effect of oxaliplatin (Figures 2J and S2). The results also displayed lower Bcl2 and higher Bax and Cleaved‐cas3 proteins in the NLRP3 silencing group (Figure 2K).

### 2.3 NLRP3/IL‐1β signaling drove PMN‐MDSCs recruitment and affected PD‐L1 expression

**FIGURE 3:**
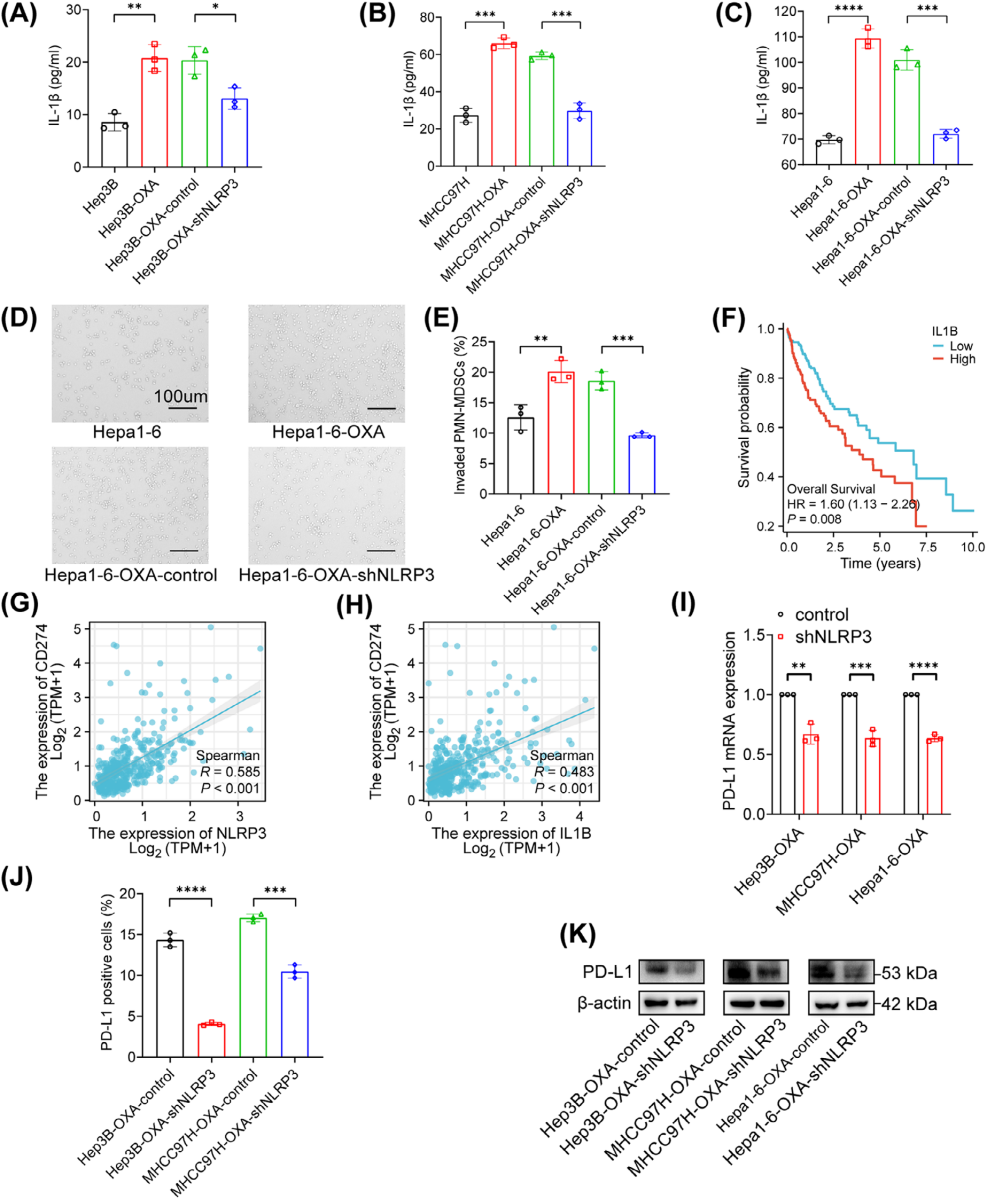
NLRP3/IL‐1β signaling drove PMN‐MDSCs recruitment and affected PD‐L1 expression. (A) The IL‐1β protein in conditional media (CM) from Hep3B, Hep3B‐OXA, Hep3B‐OXA‐control, and Hep3B‐OXA‐shNLRP3 was detected by ELISA. (B) The IL‐1β protein in CM from MHCC97H, MHCC97H‐OXA, MHCC97H‐OXA‐control, and MHCC97H‐OXA‐shNLRP3. (C) The IL‐1β protein in CM from Hepa1‐6, Hepa1‐6‐OXA, Hepa1‐6‐OXA‐control, and Hepa1‐6‐OXA‐shNLRP3. (D and E) MDSCs migration assays using CM from different cells. (F) The overall survival rate in HCC patients with high (*n* = 180) and low IL‐1β level (*n* = 179) using data from TCGA dataset. (G and H) Positive correlations between NLRP3/IL‐1β and CD274 (PD‐L1) using data from TCGA dataset. (I) PD‐L1 mRNA levels were significantly lower in oxaliplatin‐resistant cells transfected with NLRP3 shRNA. (J) PD‐L1 membrane expressions were downregulated after NLRP3 knockdown detected by flow cytometry. (K) PD‐L1 protein was detected in cells transfected with NLRP3 shRNA. *p < 0.05, **p < 0.01, ***p < 0.001.

Previously, we confirmed that IL‐1β expression was significantly reduced after NLRP3 knockdown. In the supernatant of oxaliplatin‐resistant cells, we observed a significantly higher level of IL‐1β. Conversely, when NLRP3 expression was suppressed, the IL‐1β in the supernatant of was decreased (Figures 3A–C). Our previous study showed oxaliplatin‐resistant tumors had increased accumulation of PMN‐MDSCs and decreased CD8^+^ T cells, while there was no significant difference in the frequency of tumor‐associated macrophages (TAMs).^17^ So we aimed to investigate whether the NLRP3/IL‐1β signaling pathway was responsible for the recruitment of PMN‐MDSCs. In this study, oxaliplatin‐resistant HCC cells exhibited a stronger chemotactic ability to PMN‐MDSCs. Various chemokines influence PMN‐MDSCs migration into tumors. Therefore, we speculated whether IL‐1β could mediate the migration of PMN‐MDSCs. After NLRP3 knockdown, we found that the ability of the supernatant to induce chemotaxis in PMN‐MDSCs was significantly decreased (Figures 3D and E).

In our previous study, programmed cell death 1 ligand 1 (PD‐L1) was abnormally elevated in oxaliplatin‐resistant HCC strains.^17^ However, the positive correlations between NLRP3 and PD‐L1 and IL‐1β and PD‐L1 were confirmed in HCC patients in the TCGA dataset (Figures 3G and H). In addition, high IL‐1β was associated with poorer overall survival of HCC patients (Figure 3F). In addition to the previously confirmed association of NLRP3/IL‐1B with the recruitment of MDSCs, we found that PD‐L1 expression was markedly reduced when NLRP3 was silenced (Figures 3I and J). The expression of PD‐L1 on the cell surface was significantly decreased after NLRP3 knockdown (Figure 3K). Therefore, high NLRP3 expression affected the PD‐L1 expression and PMN‐MDSC recruitment by secreting IL‐1β.

### 2.4 NLRP3/IL‐1β facilitated oxaliplatin‐resistant HCC progression in vivo

**FIGURE 4:**
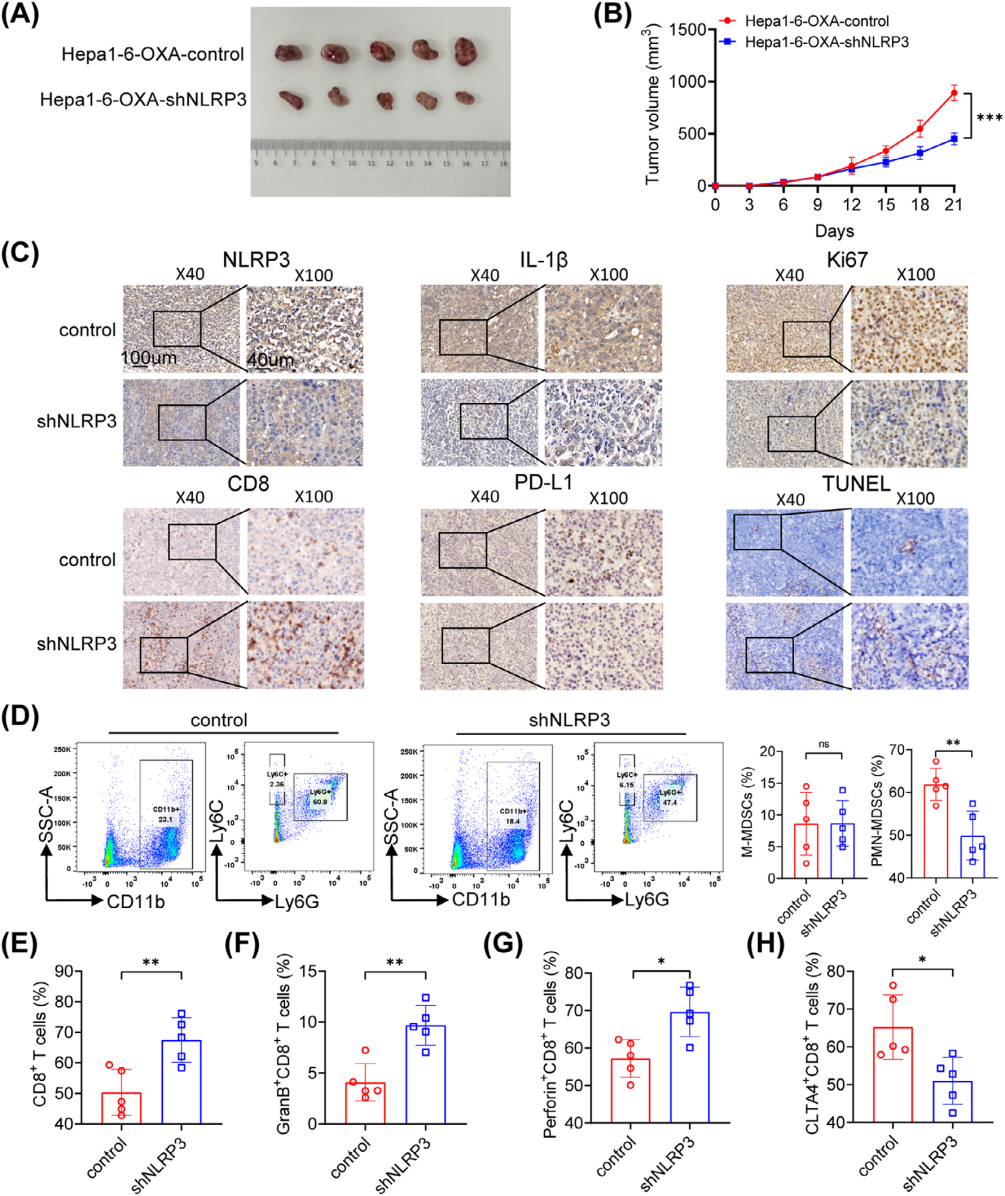
NLRP3/IL‐1β facilitated oxaliplatin‐resistant HCC progression in vivo. (A) The appearance of the subcutaneous tumors from Hepa1‐6‐OXA‐control and Hepa1‐6‐OXA‐shNLRP3 group. (B) Tumor growth curves of each group. (C) IHC staining of NLRP3, IL‐1β, Ki67, CD8, PD‐L1, and CD11b. (D) There was a significant reduction in CD11b^+^Ly6G^+^ PMN‐MDSCs level in the Hepa1‐6‐OXA‐shNLRP3 group, while the changes of CD11b^+^Ly6C^+^ M‐MDSCs showed no statistical significance. (E) The CD3^+^CD8^+^ T cells was enriched in the Hepa1‐6‐OXA‐shNLRP3 group. (F–H) The granzyme B, Perforin, and CTLA4 positive CD8^+^ T cells are depicted. *p < 0.05, **p < 0.01, ***p < 0.001.

We inoculated C57BL/6 mice with Hepa1‐6‐OXA‐shNLRP3 and Hepa1‐6‐OXA‐control cells to establish the mice model. Following the administration of oxaliplatin, there was a notable decrease in the growth rate and tumor weight in the Hepa1‐6‐OXA‐shNLRP3 group (Figures 4A and B). Furthermore, the immunohistochemical results showed that NLRP3 silencing suppressed IL‐1β, Ki67, and PD‐L1 expression and promoted CD8 expression (Figure 4C). Increasing evidence suggests MDSCs play an important immunosuppressive role in the immune microenvironment. We speculated that NLRP3/IL‐1β might be involved in the recruitment of PMN‐MDSCs to oxaliplatin‐resistant HCC. Flow cytometry analysis confirmed that NLRP3 knockdown reduced the number of PMN‐MDSCs but increased CD8^+^ T cells in the TME. In addition, the proportions of GranB^+^CD8^+^ T cells and Perforin^+^CD8^+^ T cells also increased in the Hepa1‐6‐OXA‐shNLRP3 group, whereas the proportions of CLTA4^+^CD8^+^ T cells decreased (Figures 4D–H).

### 2.5 MCC950 affected PMN‐MDSC recruitment and inhibited PD‐L1 expression

**FIGURE 5:**
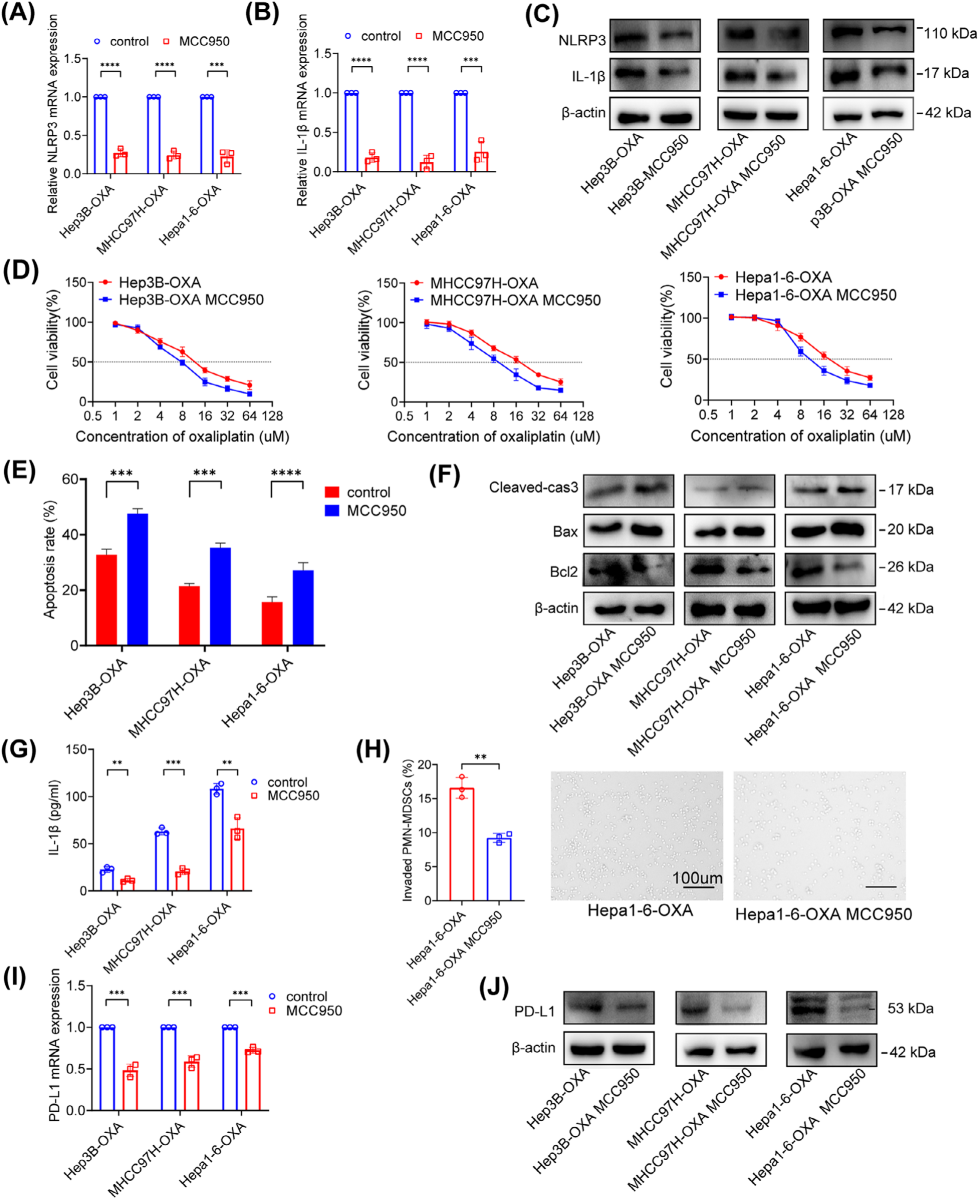
MCC950 affected PMN‐MDSC recruitment and inhibited PD‐L1 expression. (A and B) The mRNA expression of NLRP3 and IL‐1β was reduced in oxaliplatin‐resistant HCC (Hep3B‐OXA, MHCC97H, and Hepa1‐6‐OXA) cells treated with MCC950. (C) NLRP3 and IL‐1β proteins were lower in oxaliplatin‐resistant cells treated with MCC950. (D) The IC50 values of oxaliplatin‐resistant HCC cells treated with MCC950 were significantly lower than those treated with PBS. (E) The proapoptotic effect of MCC950 on oxaliplatin‐induced apoptosis was confirmed by flow cytometry in oxaliplatin‐resistant HCC cells. (F) Cleaved‐cas3, bcl2, and bax proteins in indicated cells exposed to MCC950 or PBS. (G) The IL‐1β protein in CM from indicated cells treated with MCC950 or PBS was detected by ELISA. (H) MDSC migration ability was tested utilizing CM from Hepa1‐6‐OXA cells exposed to MCC950 or PBS. (I) PD‐L1 mRNA was reduced in indicated cells treated with MCC950. (J) The PD‐L1 protein was lower in indicated treatment groups. *p < 0.05, **p < 0.01, ***p < 0.001.

MCC950 is a specific small molecule inhibitor of NLRP3. The results unequivocally demonstrated that treatment with a 10 μM dose of MCC950 substantially decreased the expression of NLRP3 and IL‐1β (Figures 5A–C). The Cell Counting Kit‐8 (CCK‐8) assay showed that the IC50 of oxaliplatin decreased after MCC950 treatment (Figure 5D). The effect of MCC950 on apoptosis was also evaluated. Flow cytometry analysis displayed that MCC950 promoted the proapoptotic effects of oxaliplatin (Figures 5E and S3). It also demonstrated lower Bcl2 and higher Bax and Cleaved‐cas3 proteins in the NLRP3 silencing group (Figure 5F). The levels of IL‐1β in supernatants were significantly lower in oxaliplatin‐resistant HCC cells after MCC950 treatment (Figure 5G). On the one hand, oxaliplatin‐resistant HCC cells showed a significantly decreased ability to chemotactically induce PMN‐MDSCs after MCC950 treatment (Figure 5H). On the other hand, we observed remarkably lower PD‐L1 expression in the MCC950 group (Figures 5I and J). Thus, MCC950 affected both PMN‐MDSC recruitment and PD‐L1 expression in vitro.

### 2.6 IL‐1β blocking inhibited PMN‐MDSCs recruitment and enhanced CD8^+^T cell‐killing ability

**FIGURE 6:**
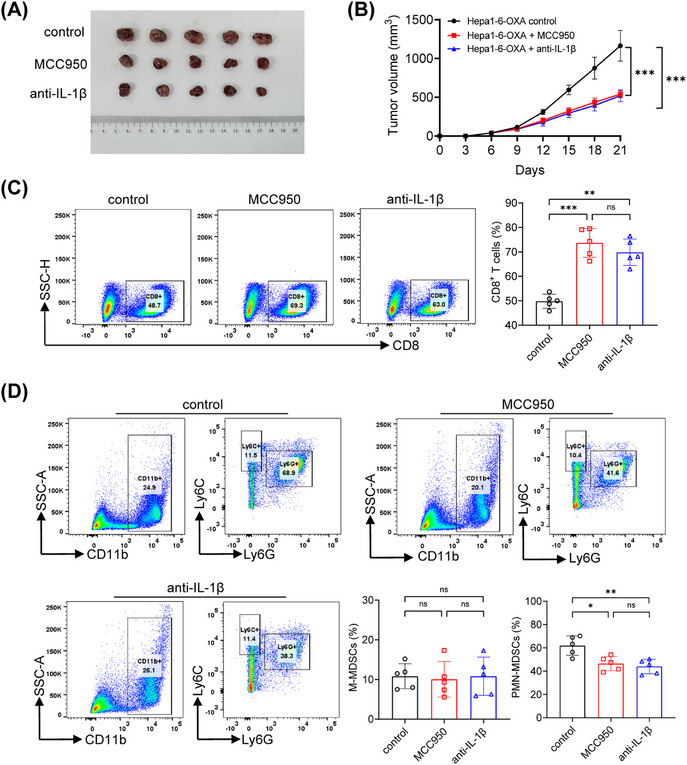
IL‐1β blocking inhibited PMN‐MDSCs recruitment and enhanced CD8^+^T cell‐killing ability. (A and B) The tumor appearance and growth curves were shown in different group. (C) The infiltration of CD3^+^CD8^+^ T cells was much higher in mice treated with MCC950 or anti‐IL‐1β. (D) A significant reduction of CD11b^+^Ly6G^+^ PMN‐MDSCs infiltration when treated with MCC950 or IL‐1β antibody, while the changes in CD11b^+^Ly6C^+^ M‐MDSCs showed no statistical significance. *p < 0.05, **p < 0.01, ***p < 0.001.

To evaluate the effect of IL‐1β in tumor growth, we further used the IL‐1β antibody and MCC950 for in vivo studies. Indeed, IL‐1β antibody or MCC950 treatment significantly delayed tumor progression (Figures 6A and B). Our observations revealed that both IL‐1β antibody and MCC950 treatment resulted in a significant reduction in the proportions of PMN‐MDSCs in the Hepa1‐6‐OXA‐control group. However, the M‐MDSCs infiltration was not affected by these interventions. In addition, the IL‐1b antibody and MCC950 significantly increased the percentage of CD8^+^ T cells in tumor‐bearing mice (Figures 6C and D). These results suggested that IL‐1β blocking by the IL‐1β antibody or MCC950 could reverse immunosuppression by inhibiting PMN‐MDSCs recruitment and enhancing CD8^+^ T cell‐killing ability.

### 2.7 IL‐1β blocking enhanced the effect of anti‐PD‐L1 therapy

**FIGURE 7:**
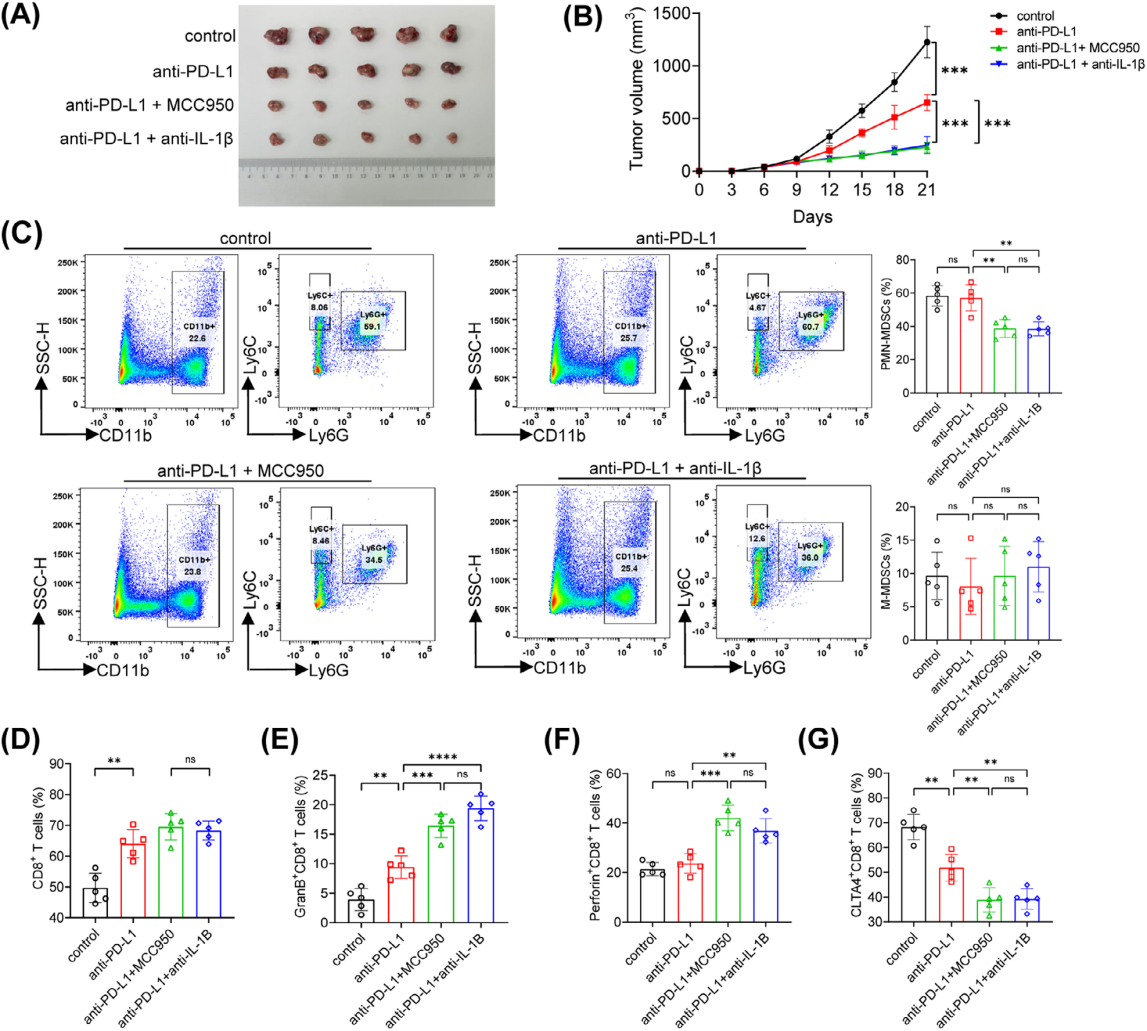
IL‐1β blocking enhanced the effect of anti‐PD‐L1 therapy. (A and B) The tumor appearance and growth curves were shown in different group. (C–G) The proportions of MDSCs, CD8^+^ T cells, along with the levels of granzyme B, Perforin, and CTLA4 positive CD8^+^ T cells are displayed. *p < 0.05, **p < 0.01, ***p < 0.001.

The PD‐L1 antibody is one of the immunotherapy drugs for HCC, but its efficiency is only 10−30% when used alone. Our study showed that the IL‐1β antibody and MCC950 inhibited the chemotaxis of PMN‐MDSCs in vitro. In oxaliplatin‐resistant HCC, there was an upregulation of PD‐L1, which directly hindered the function of cytotoxic T cells. Next, we proceeded to explore the potential synergistic effect of the PD‐L1 blockade in enhancing the antitumor immune response by IL‐1β blocking. Our findings indicated that treatment with the PD‐L1 antibody alone effectively inhibited the progression of oxaliplatin‐resistant HCC. However, the antitumor effect was notably enhanced when the PD‐L1 antibody was administered in combination, as depicted in Figures 7A and B.

A substantial reduction in the infiltration of PMN‐MDSCs and an increased frequency of CD8^+^ T cells were also observed in the combined therapy group, and mice treated with the combined therapy significantly increased the proportions of GranB^+^CD8^+^ T cells and Perforin^+^CD8^+^ T cells (Figures 7C–G).

## 3 DISCUSSION

The TME comprises various types of immunosuppressive cells, such as regulatory T cells (Tregs) and MDSCs, leading to the evasion of tumor immune surveillance. Recent studies have emphasized the critical role of inflammasomes in promoting tumor progression in TME.^25^ This study demonstrated that NLRP3/IL‐1β contributed to oxaliplatin resistance and mediated an immunosuppressive microenvironment in HCC.

The NLRP3 signaling pathway is critical for the generation of IL‐1β, which subsequently activates immune cells and triggers the production of cytokines. In a previous study, after vaccination with dendritic cells, the survival of bearing melanoma xenograft NLRP3‐deficient mice was significantly prolonged due to the reduced number of MDSCs.^26^ Bruchard et al.^27^ found that gemcitabine and 5‐fluorouracil could activate the NLRP3 inflammasome to secrete IL‐1β, thus preventing CD4^+^ T cells form producing IL‐17. In NLRP3‐deficient mice, the growth and metastasis of breast tumor cells were inhibited by reduced MDSCs infiltration.^28^ The NLRP3 signaling pathway promoted the expansion of immunosuppressive macrophages, thereby promoting tumor growth in pancreatic ductal adenocarcinoma.^29^ The above studies suggested that the inhibition of NLRP3 can reduce the immunosuppressive cells’ number and enhance antitumor immunity.

The interactions between cancer cells and their surrounding environments plays a crucial role in cancer progression and drug resistance. Bidirectional communication is widely recognized as a significant factor in these processes. However, the tumor‐promoting role and specific mechanism of NLRP3 in the HCC microenvironment have not yet been fully elucidated. In this study, the IC50 values of oxaliplatin and subcutaneous tumor volume were significantly reduced after NLRP3 knockdown or treatment with MCC950. Our findings provided evidence that the recruitment of PMN‐MDSCs, partly mediated by IL‐1β, contributes to oxaliplatin insensitivity.

During the early stages of differentiation, PMN‐MDSCs are composed of granulocytes, monocytes, macrophages, and myeloid progenitors.^30^, ^31^, ^32^ Typically, MDSCs differentiate into dendritic cells, macrophages, and granulocytes. However, upon stimulation by various factors, MDSCs are mobilized to peripheral blood, liver, spleen, lymphatic system, and other organs. Multiple studies have provided evidence that MDSCs mediate immunosuppression of T lymphocytes.^32^ First, MDSCs can promote the proliferation of Tregs while inhibiting the proliferation of CD8^+^ T cells. Second, MDSCs can directly differentiate into M2 TAMs, resulting in T cell apoptosis and immunosuppression. Furthermore, MDSCs secrete IL‐10 and TGF‐β, which inhibit the immune‐mediated cytotoxicity of cytotoxic T lymphocytes.^33^ In the current study, we showed the effect of NLRP3/IL‐1β on PMN‐MDSCs recruitment. Treatment with IL‐1β antibody or MCC950 lead to a notable decrease in the recruitment of PMN‐MDSCs. Concurrently, there was an increase in the proportions of IFN‐γ^+^CD8^+^ T cells and GranB^+^CD8^+^ T cells, leading to partial restoration of the killing function of T cells. Hence, targeting the NLRP3/IL‐1β pathway emerges as a promising therapeutic approach for HCC.

PD‐L1 serves as a crucial checkpoint in immunosuppression, leading to T cell exhaustion and immune tolerance, and is recognized as a major contributor to tumor immune evasion.^34^, ^35^ Moreover, certain factors can reinforce the PD‐L1 level and hinder the activation of immune effector cells by modulating PD‐L1 expression in the TME. However, until now, the relationship between NLRP3 and PD‐L1 expression has not been thoroughly investigated in HCC. Hence, we speculated that NLRP3 might exert regulatory control over PD‐L1. Our findings demonstrated a significant reduction in PD‐L1 expression upon NLRP3 downregulation. In vivo experiments further revealed that inhibition of the NLRP3/IL‐1β axis could reverse the immunosuppressive TME and enhance the cytotoxic activity of CD8^+^ T cells. Additionally, our results indicated that the combined treatment of MCC950 or IL‐1β antibody with PD‐L1 antibody yielded greater benefits than the PD‐L1 monoclonal antibody alone. This study provides evidence that elevated NLRP3/IL‐1β expression promoted the progression of oxaliplatin‐resistant HCC by inducing PD‐L1 upregulation as well as recruitment of PMN‐MDSCs. Hence, we proposed NLRP3 as a promising therapeutic target for overcoming oxaliplatin resistance and reshaping the TME in HCC.

However, it is important to acknowledge certain limitations of this study. First, we utilized shRNA‐mediated knockdown of NLRP3 rather than employing CRISPR/Cas9 gene editing, despite achieving efficient knockdown. Second, while existing studies explored the relationship between NLRP3 and other chemotherapy drugs, our research lacks a little direct literature support but is innovative.

In summary, our findings indicated that elevated levels of NLRP3/IL‐1β in tumor cells contribute to oxaliplatin resistance and tumor progression through the modulation of PMN‐MDSC recruitment and PD‐L1 expression. The downregulation of NLRP3/IL‐1β can greatly enhance the efficacy of oxaliplatin chemotherapy. A thorough understanding of the reciprocal communication between tumor cells and PMN‐MDSCs, as well as the upstream regulators of PD‐L1, can offer more effective approaches for cancer treatment.

## 4 MATERIALS AND METHODS

### 4.1 Cell lines

The HCC cell lines Hep3B, MHCC97H, and Hepa1‐6 were acquired from Zhongshan Hospital. To establish oxaliplatin‐resistant strains, Hep3B‐OXA, MHCC97H‐OXA, and Hepa1‐6‐OXA, we followed the protocol described in our previously published article.^36^, ^37^ HCC cells were initially plated in T25 cell culture flasks for 1 day. Following this, the medium was substituted with minimum essential medium or Dulbecco's modified Eagle's medium supplemented with 10% fetal bovine serum (FBS) (Gibco) and treated with 2 μM oxaliplatin (Sigma). After 2 days, the treatment was discontinued, allowing the cells to recovery. Once the surviving cell population reached 80% confluence, the cells were passaged and subjected to another 2 days of oxaliplatin treatment. This process was repeated, and the concentrations of oxaliplatin was gradually increased, until the cells exhibited stable resistance up to 25 μM oxaliplatin. The IC50 values of HCC oxaliplatin resistant cells and the parental cells were displayed in Figure S1.

### 4.2 Cell viability assay

To assess the cytotoxicity and resistance to oxaliplatin, we employed the CCK‐8 (Yeasen). The cells were seeded into 96‐well plates at a density of 3 × 10^3^ cells/well. After incubation for 12 h, oxaliplatin was added at different concentrations for a duration of 48 h. After the treatment period, 10 μL of CCK‐8 reagent was added and incubated for 2 h. The optical density was measured at 450 nm.

### 4.3 Lentivirus vector preparation and transfection

shRNA sequences targeting NLRP3 were designed and synthesized by GenePharma (Genomeditech). After 72 h of transfection, the cells were subjected to selection in medium containing 6 μg/mL puromycin for 48 h.

### 4.4 Quantitative real‐time PCR

Total RNA was extracted and cDNA was acquired according to the manufacturer's instructions (Thermo Scientific). qPCR was performed using SYBR Green Master Mix (EZBioscience) according to the instructions for the PCR amplifier (Bio‐Rad).

### 4.5 Western blot

Proteins were extracted and separated by sodium dodecyl sulfate‐polyacrylamide gel electrophoresis and transferred onto polyvinylidene fluoride membranes (Millipore). The primary antibodies were incubated overnight at 4°C and the secondary antibodies were then incubated for 1 h at room temperature. The enhanced chemiluminescence (Yeasen) was used for visualization.

### 4.6 Enzyme‐linked immunosorbent assay

Cell culture supernatants were collected and centrifuged for 10 min at 250*g*. The levels of various cytokines were measured by Enzyme linked immunosorbent assay (ELISA) kits (R&D Systems) following the manufacturer's protocols.

### 4.7 Immunohistochemistry

Primary antibodies against CD8, PD‐L1, Ki67, and 3,3′‐diaminobenzidine (Abcam) were used for immunohistochemistry (IHC) by the avidin–biotin–peroxidase complex method.

### 4.8 Flow cytometry

The tumor tissues were sectioned into smaller pieces and subjected to enzymatic digestion using collagenase IV (1 mg/ml) at a temperature of 37°C for a duration of 1 h. The resultant cell suspension was then treated with Fc antibody to block and then stained with antibody. All antibodies were purchased from BioLegend (BioLegend).

### 4.9 MDSC isolation by magnetic bead cell sorting

CD11b^+^Ly6G^+^ PMN‐MDSCs were isolated using an isolation kit (Miltenyi Biotec). Following FcR blocking, anti‐Ly6G‐biotin antibody and antibiotin microbeads were used for staining. The cell suspension was then processed by passing it through an LS separation column, resulting in the retention of our target cells.

### 4.10 MDSC transwell migration assay

To assess the migration of MDSCs, we utilized 24‐well plates with transwell polycarbonate‐permeable supports (8.0 μm). Fresh PMN‐MDSCs (1.5 × 10^6^) were seeded in the upper chambers. The conditioned media (CM) from Hepa1‐6, Hepa1‐6‐OXA, Hepa1‐6‐OXA‐control, and Hepa1‐6‐OXA‐shNLRP3 cells were placed in the lower chamber. Following a 48 h incubation period, the number of migrated MDSCs in the bottom compartment was determined.

### 4.11 Animal experiments

C57BL/6J mice (5−7 weeks old, weighing 20–29 g) were obtained from Shanghai SLAC Laboratory. This study conformed to the provisions of the Declaration of Helsinki. 2.5 × 10^6^ Hepa1‐6 cells were inoculated subcutaneously into the right flank of the mice. Seven days after injection, oxaliplatin at a dosage of 5 mg/kg was administrated twice within 1 week. And anti‐PD‐L1 antibody (200 μg, intraperitoneally, q3d; Bio X cell), anti‐IL‐1β antibody (50 μg, intratumorally, q3d; Bio X cell), MCC950 (15 mg/kg, intraperitoneally, q3d; Selleck) were also used.

## AUTHOR CONTRIBUTIONS

L. W. and Y. X. designed the project, analyzed the results, and wrote the manuscript. L. W., Z. F., and Q. B. performed the related experiments. All statistical analyses were performed by L. W., Z. F., Q. B., Y. F., and C. R., Y. X., and R. Z. reviewed the manuscript. All the authors have read and approved the final manuscript.

## CONFLICT OF INTEREST STATEMENT

The authors report no conflict of interest in this work.

## ETHICS STATEMENT

All animal experiments conducted in this study were approved by the Animal Care Committee and the Ethical Committee on Animal Experiments of Zhongshan Hospital, Fudan University (Permit Number: B2022‐164) were performed in accordance with the guidelines of the Shanghai Medical Experimental Animal Care Commission.

## Supporting information

Supporting Information

## Data Availability

The data that support the findings of this study are available from the corresponding author upon reasonable request.
