# Supplementary material for: NLRP3/IL‐1β induced myeloid‐derived suppressor cells recruitment and PD‐L1 upregulation promotes oxaliplatin resistance of hepatocellular carcinoma
Source: MedComm (2020). 2023 Dec 19;4(6):e447. doi: 10.1002/mco2.447 (PMC10728756; doi:10.1002/mco2.447)
Supplement: Supplementary file 1 — Supporting Information [file MCO2-4-e447-s001.docx]

**NLRP3/IL-1β induced myeloid-derived suppressor cells recruitment and PD-L1 upregulation promotes oxaliplatin resistance of hepatocellular carcinoma**

Wenfeng Liu^1,2,#^, Feng Zhang^1,2,#^, Bing Quan^1,2^, Fan Yao^1,2^, Rongxin Chen^1,2^, Zhenggang Ren^1,2^, Xin Yin^1,2*^

^1^Liver Cancer Institute, Zhongshan Hospital, Fudan University, Shanghai 200032, China

^2^National Clinical Research Center for Interventional Medicine, Shanghai 200032, China

^#^Wenfeng Liu and Feng Zhang contributed equally to this work.

^*^**Address Correspondence to:**

Professor Xin Yin, 136 Yi Xue Yuan Road, Shanghai 200032, China. Email: [yin.xin@zs-hospital.sh.cn](mailto:yin.xin@zs-hospital.sh.cn)

**Table S1.** Primers used for quantitative real-time PCR in this study.

**Table S2.** Antibodies lists for the experiments.

**Table S3.** Antibodies used for flow cytometry.

**Figure S1.** IC50 evaluation on HCC oxaliplatin resistant cells and their parental cells.

**Figure S2.** The proapoptotic effect of NLRP3 silencing on oxaliplatin-induced apoptosis was confirmed by flow cytometry in oxaliplatin-resistant HCC cells.

**Figure S3.** The proapoptotic effect of MCC950 on oxaliplatin-induced apoptosis was confirmed by flow cytometry in oxaliplatin-resistant HCC cells.

**Figure S4.** IC50 evaluation on HCC cells treated with and without NLRP3 agonist BMS-986299 (2 μM).

**Figure S5.** In vivo cytotoxicity measurement of MCC950 and IL-1β antibody. (A) Blood biochemical test in mice intravenously treated with MCC950 and IL-1β antibody. (B) Pathological analysis of major organs in mice intravenously. Data presented as mean±SD (n=5). Statistical differences were determined using unpaired student’st-tests.

**Table S1. Primers used for quantitative real-time PCR in this study.**

| **Gene name** | **Forward Sequence (****5’ to 3’)** | **Forward Sequence (5’ to 3’)** |
| --- | --- | --- |
| Human β-Actin | CATGTACGTTGCTATCCAGGC | CTCCTTAATGTCACGCACGAT |
| Human NLRP3 | GGACTGAAGCACCTGTTGTGCA | TCCTGAGTCTCCCAAGGCATTC |
| Human IL-1β | TTCGACACATGGGATAACGAGG | TTTTTGCTGTGAGTCCCGGAG |
| Human PD-L1 | TGGCATTTGCTGAACGCATTT | TGCAGCCAGGTCTAATTGTTTT |
| Mouse β-Actin | GGCTGTATTCCCCTCCATCG | CCAGTTGGTAACAATGCCATGT |
| Mouse NLRP3 | CTCCAACCATTCTCTGACCAG | ACAGATTGAAGTAAGGCCGG |
| Mouse IL-1β | GGTCAAAGGTTTGGAAGCAG | TGTGAAATGCCACCTTTTGA |
| Mouse PD-L1 | GCTCCAAAGGACTTGTACGTG | TGATCTGAAGGGCAGCATTTC |

**Table S2. Antibodies lists for the experiments.**

| **Marker** | **Provider** | **Catalog No.** | **Concentration** |
| --- | --- | --- | --- |
| β-Actin | Biyotime | AF5003 | 1:1000 for WB |
| Goat Anti-Rabbit IgG (H+L) | Biyotime | A0277 | 1:1000 for WB |
| Goat Anti-Mouse IgG (H+L) | Biyotime | A0286 | 1:1000 for WB |
| NLRP3 | Abcam | ab263899 | 1:1000 for WB |
| IL-1β | Abcam | ab254360 | 1:1000 for WB |
| Bcl2 | Proteintech | 68103-1-Ig | 1:2000 for WB |
| Bax | Proteintech | 60267-1-Ig | 1:5000 for WB |
| Cleaved-caspase3 (Cleaved-cas3) | Abcam | ab32042 | 1:500 for WB |
| NLRP3 | Sigma | [SAB5700723](https://www.sigmaaldrich.cn/CN/zh/product/sigma/sab5700723) | 1:100 for IHC |
| IL-1β | CST | 12242 | 1:100 for IHC |
| Ki67 | Abcam | ab16667 | 1:200 for IHC |
| CD8 | Abcam | ab217344 | 1:2000 for IHC |
| PD-L1 (anti-human) | CST | 13684 | 1:1000 for WB 1:200 for IHC |
| PD-L1 (anti-mouse) | CST | 60475 | 1:1000 for WB |
| PD-L1 (anti-mouse) | CST | 64988 | 1:200 for IHC |

**Table S3. Antibodies used for flow cytometry.**

| **Antibody** | **Clone** | **Company** |
| --- | --- | --- |
| anti-CD45 | 30-F11 | BioLegend |
| anti-CD11b | M1/70 | BD |
| anti-Gr-1 | RB6-8C5 | BioLegend |
| anti-CD8 | 53-6.7 | eBioscience |
| anti-Ly6C | HK1.4 | BioLegend |
| anti-Ly6G | 1A8 | BioLegend |
| anti-CD3 | 145-2C11 | eBioscience |
| anti-CD4 | RM4-5 | eBioscience |
| anti-Granzyme B | QA16A02 | BioLegend |
| anti-CTLA4 | UC10-4B9 | BioLegend |
| anti-Perforin | S16009A | BioLegend |


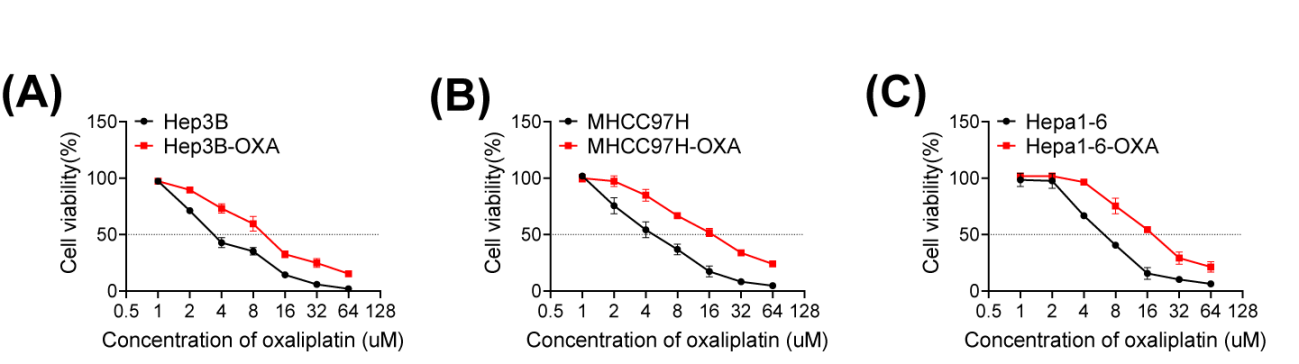


**Figure S1.** IC50 evaluation on HCC oxaliplatin resistant cells and their parental cells.


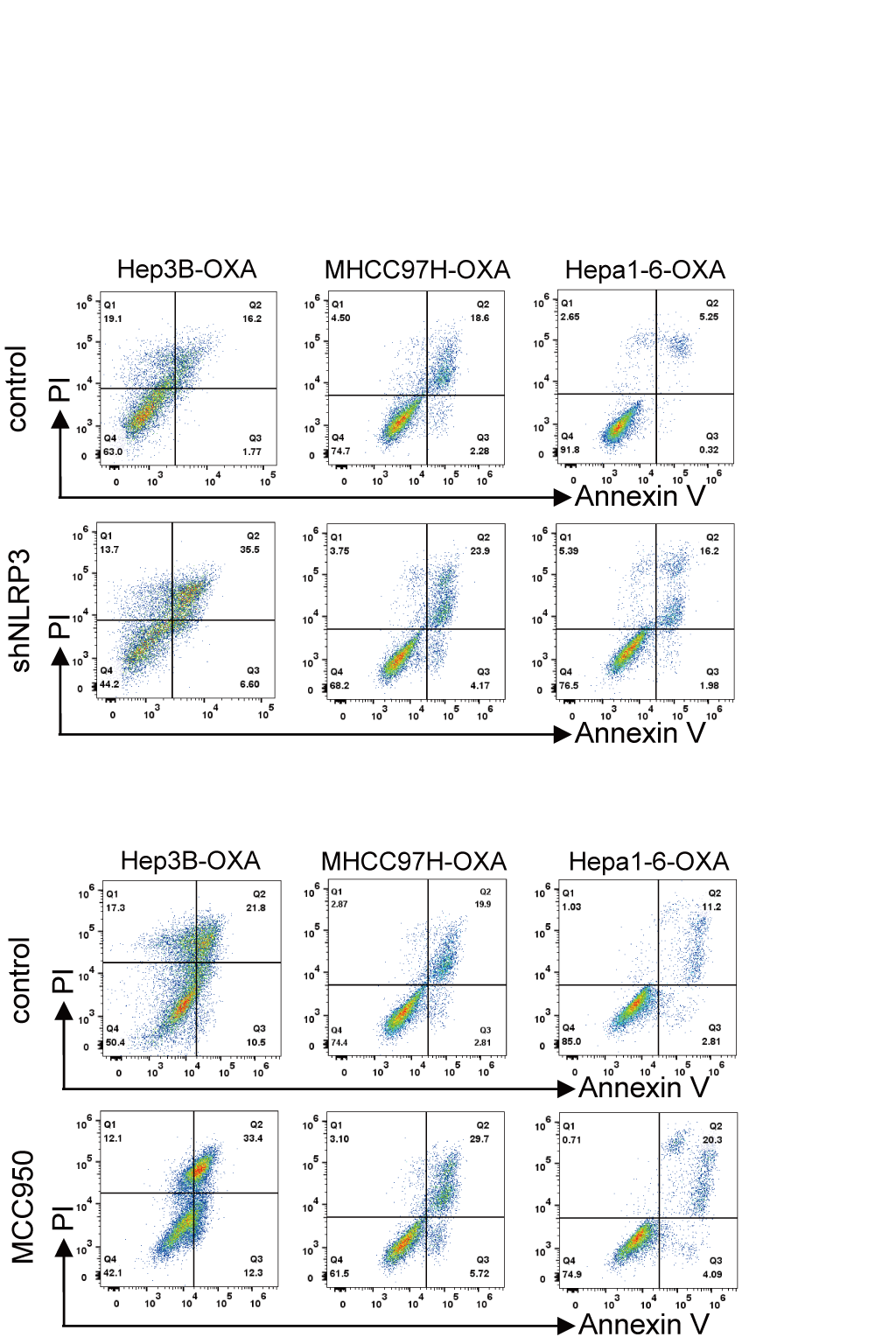


**Figure S2.** The proapoptotic effect of NLRP3 silencing on oxaliplatin-induced apoptosis was confirmed by flow cytometry in oxaliplatin-resistant HCC cells.


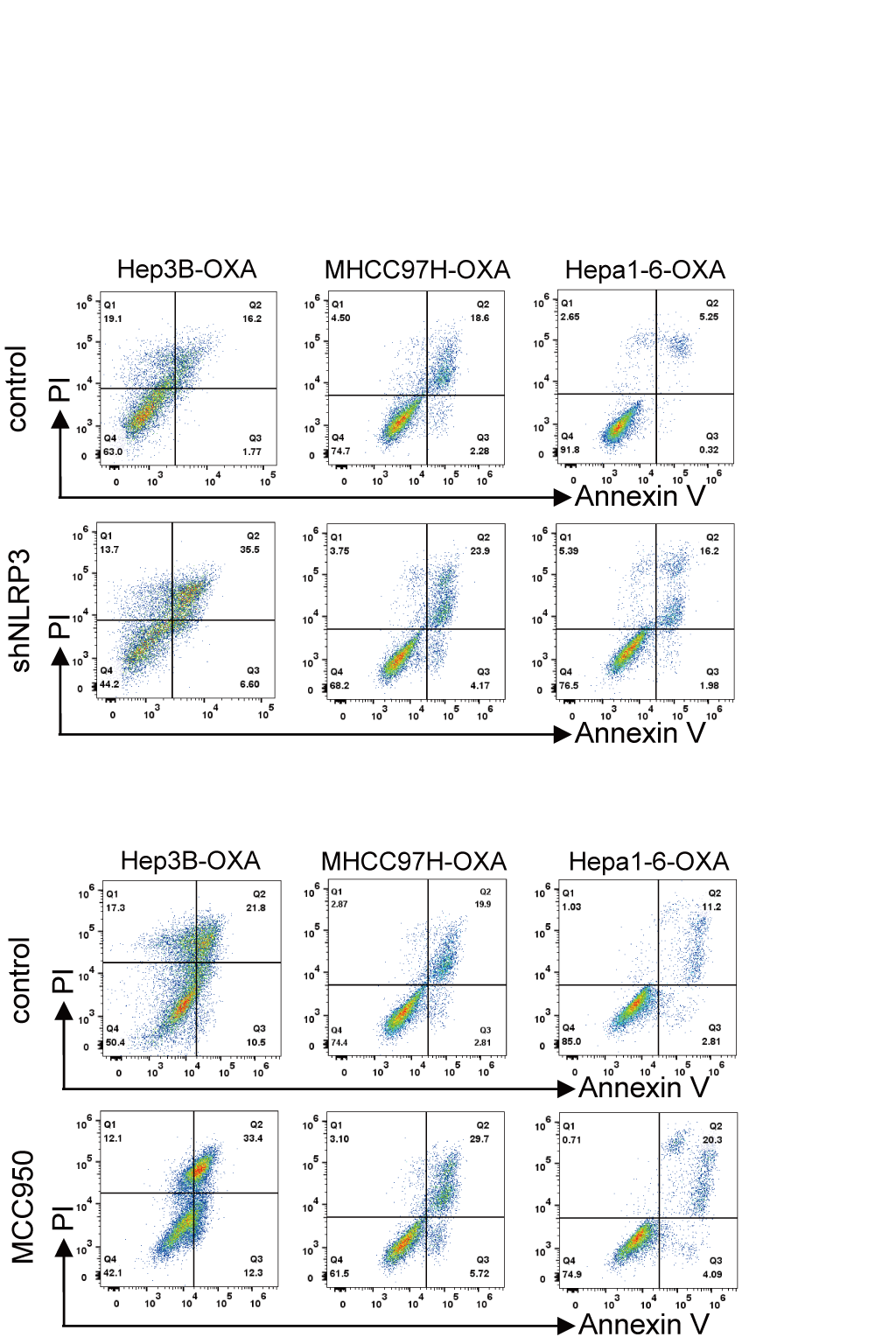


**Figure S3.** The proapoptotic effect of MCC950 on oxaliplatin-induced apoptosis was confirmed by flow cytometry in oxaliplatin-resistant HCC cells.


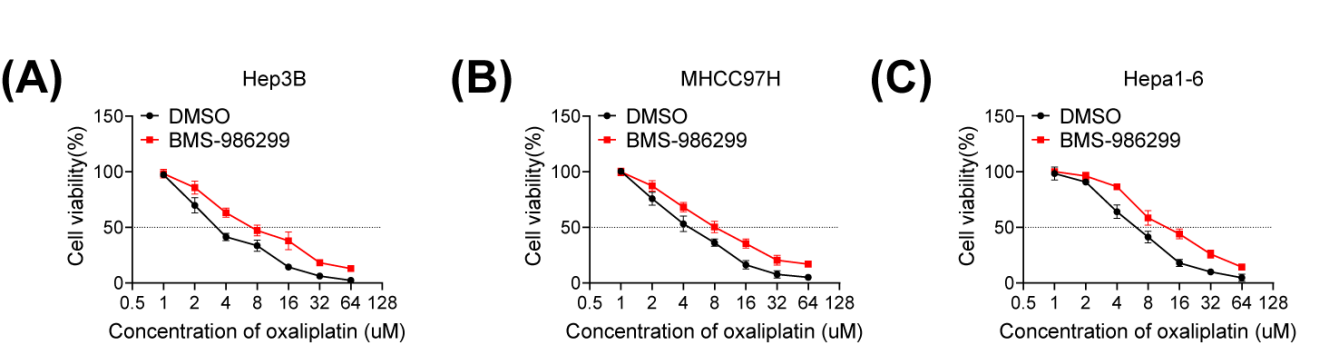


**Figure S4.** IC50 evaluation on HCC cells treated DMSO and NLRP3 agonist BMS-986299 (2 μM, MedChemExpress, United States).


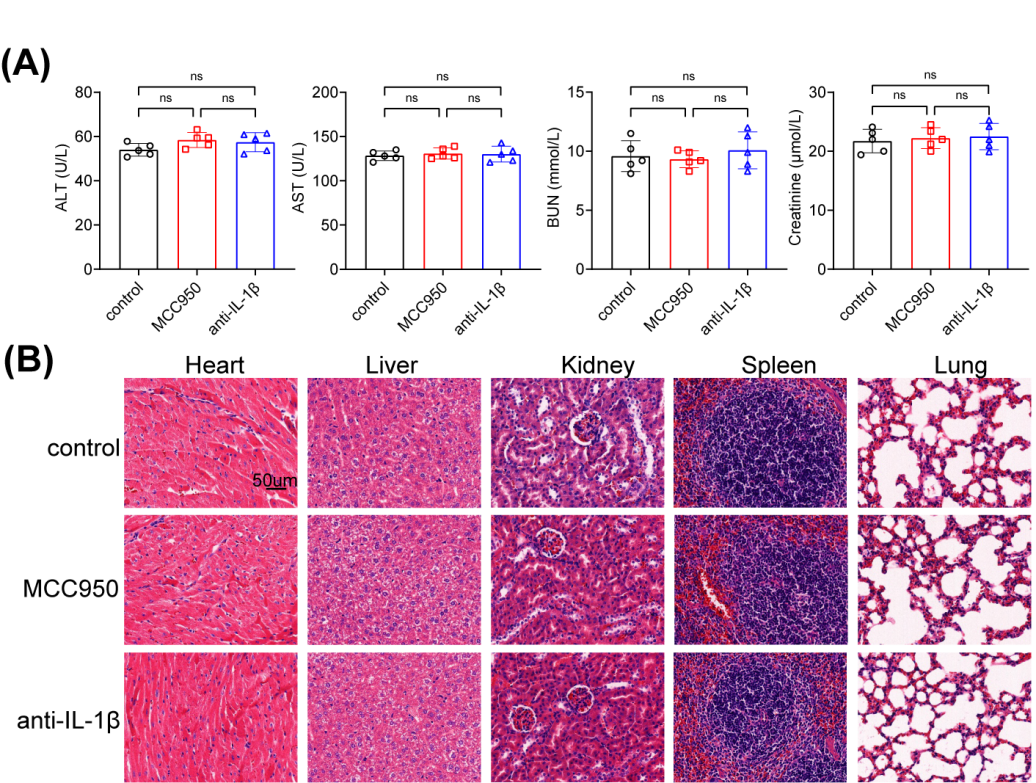


**Figure S5.** In vivo cytotoxicity measurement of MCC950 and IL-1β antibody. (A) Blood biochemical test in mice intravenously treated with MCC950 and IL-1β antibody. (B) Pathological analysis of major organs in mice intravenously. Data presented as mean±SD (n=5). Statistical differences were determined using unpaired student’st-tests.
